# Two Sides of the Same Coin: Punishment and Forgiveness in Organizational Contexts

**DOI:** 10.3389/fpsyg.2022.908021

**Published:** 2022-07-05

**Authors:** Gijs Van Houwelingen, Marius Van Dijke, Niek Hoogervorst, Lucas Meijs, David De Cremer

**Affiliations:** ^1^Amsterdam Business School, University of Amsterdam, Amsterdam, Netherlands; ^2^Rotterdam School of Management, Erasmus University, Rotterdam, Netherlands; ^3^Nottingham Business School, Nottingham Trent University, Nottingham, United Kingdom; ^4^NUS Business School, National University of Singapore, Singapore, Singapore

**Keywords:** forgiveness, punishment, moral identity, social cognitive theory, reconciliation

## Abstract

Punishment and forgiveness are two very different responses to a moral transgression that both have been argued to restore perceptions of moral order within an organization. Unfortunately, it is currently unclear what motivates organizational actors to punish or forgive a norm transgressor. We build on social cognitive theory to argue that punishment and forgiveness of a transgressor are both rooted in self-regulatory processes. Specifically, we argue that organizational actors are more likely to respond to intentional transgressions with punishment, and to unintentional transgressions with forgiveness. However, these effects of transgressor intentionality should be found in particular among actors for whom moral identity is central (vs. peripheral). We find support for these predictions in a laboratory experiment and a field study among organizational leaders. By simultaneously studying punishment and forgiveness in organizational settings, we provide crucial insight in their shared motivational bases, as well as into important differences between the two.

## Introduction

Organization members commonly transgress moral norms in the workplace. For instance, research finds that over 40% of organizational members indicate that they have over-reported the number of hours they worked, covered up incidents, misled customers, took credit for somebody else’s ideas, or lied to their supervisor (Adler, 2007; Lindsey et al., 2011; see also Grover, 1993; Prater and Kiser, 2002; Payne, 2008; Shulman, 2011; Edelman and Larkin, 2015). Transgressions like these require a response to restore a sense of moral order within the organization. If moral transgressions are ignored, employees quickly start to perceive that the organization cares little about moral standards, thus eroding perceptions of ethical leadership and a just climate.

Scholars commonly suggest two responses that may induce perceptions among members that the moral order within the organization is restored in the aftermath of a transgression—depending on whether the perceived motives for a forgiving or punitive response align with transgressor motives (Gollwitzer and Okimoto, 2021). Specifically, moral transgressors may be punished for their transgression, or they may be forgiven. On the face of it, these two responses are very different from each other: whereas punishment refers to the infliction or imposition of a penalty as retribution for an offence, forgiveness implies that the victim will not seek (further) revenge or reparations (Enright, 1991; Baumeister et al., 1998). However, more recent research has shown that punishment and forgiveness may be more similar than it appears, both in terms of motivations as well as in terms of consequences (Strelan, 2017; Strelan et al., 2017). Unfortunately, it is unclear what makes organizational actors decide to punish transgressions of moral norms, and what makes them forgive the transgressor.

In this paper we build on social cognitive theory (Bandura, 1991, 2011) to argue that the decisions to punish or forgive a transgressor are both rooted in self-regulatory processes. Social cognitive theory describes human behavior, including morally motivated behavior, in terms of self-regulatory systems that underlie setting goals and striving towards these goals. Based on this theory, we identify transgressor intentionality (Leunissen et al., 2013) and moral identity centrality (Aquino et al., 2009; Boegershausen et al., 2015) as crucial antecedents of punishment and forgiveness. Transgressor intentionality refers to whether the transgressor wanted the transgression to happen or, conversely, accidentally transgressed a moral norm; moral identity refers to the centrality of moral beliefs to the self-concept (Bandura, 1991, 2002). Intentional transgressions are likely to be punished, whereas unintentional transgressions are more likely to be forgiven. We will argue, however, that these effects of transgressor intentionality on punishment and forgiveness should be found in particular among actors for whom moral identity is central; actors for whom moral identity is peripheral will care less about punishment and forgiveness of moral transgressions.

In using social cognitive theory to integrate the disconnected literatures on punishment and on forgiveness, we seek to offer contributions to various literatures. First, although organizational scholars have studied punishment for decades, they almost always focused on effects of punishment on performance (Podsakoff et al., 2006; McNamara et al., 2022). The few studies on punishment of moral transgressions focused on the interests of the authority or the organization as antecedents of such punishment (Hoogervorst et al., 2010; Cramwinckel et al., 2013; Desmet et al., 2015; Van Houwelingen et al., 2020). This paper begins to develop our understanding of how moral motivations shape punishment of moral transgressions in organizations. Second, research has shown that the extent to which morality is a central concern for a person (i.e., moral identity centrality) can be associated with higher levels of punitiveness (Hofmann et al., 2018), but also with a stronger preparedness to forgive and weaker inclinations to punish (e.g., Aquino et al., 2007). In the present research we show for what type of transgressions, moral identity centrality is likely to strengthen punitive tendencies (i.e., intentional transgressions), and for which type of transgressions, moral identity is likely to strengthen forgiveness (i.e., unintentional transgressions).

## Punishment, Forgiveness, and the Moral Order

Research suggests that moral transgressions are experienced quite literally as intrusions upon a moral order that needs to be set right (Cushman, 2015; Strelan et al., 2017). This is because people tend to see moral norms and rules as touchstones, helping them make sense of the social world and make it more predictable (Fitness, 2001; Fitness and Peterson, 2008). A transgression of a moral norm thus often leaves people feel uncertain and uneasy about their social environment (Funk et al., 2014). As a result, they feel the need to be reassured that the moral norms are still in place and that other people are still likely to behave in line with these norms (Fitness, 2001).

Research on motivations underlying punitive judgments and decisions has mainly focused on lay-people (e.g., in the mock-jury literature; Mazzella and Feingold, 1994) or professional court judges (e.g., Danziger et al., 2011). Much of this research has shown that punitive decisions can be driven by moral motives, such as a desire to restore moral order (Carlsmith et al., 2002). Specifically, an important function of punishment is to have a transgressor pay for his/her actions by incurring suffering (Carlsmith, 2006; Fitness and Peterson, 2008). In support of this analysis of the function of punishment, research shows that some transgressors *want* to be punished for their misdeeds, as they feel they would not be able to move on otherwise (Fitness, 2001). This is also true on the victim- and observer-side: Punishment has been found to be empowering to victims (Strelan et al., 2020), and allows all parties to move on beyond the transgression (Strelan et al., 2017). Other research shows that people often use the norm of proportionality to assess whether or not punishment is justified: Only when the suffering caused by punishment is seen as equivalent to the harm caused, is punishment considered to be sufficient (Skarlicki and Kulik, 2004).

Forgiveness, in contrast, involves remittance of the moral debt brought about by the transgression (McCullough et al., 2013): It allows both transgressor and victim to restore the relationship and move on (Fitness and Peterson, 2008). In line with this, withholding forgiveness has been shown to be a powerful motivator for the transgressor to engage in reconciliatory behaviors (Zheng et al., 2016). Forgiveness is much more than letting go of a grudge, but is, instead a complex intra- and interpersonal process that involves changes in attitudes as well as emotions and behaviors (Forster et al., 2020). While granting forgiveness may at first sight seem to be solely in the interest of the transgressor, forgiveness also performs important functions for the victim. Forgiving a perpetrator allows a victim to let go of negative feelings towards the transgressor, and to no longer having to define themselves and the relation in terms of the harm incurred (Fitness, 2001; Fitness and Peterson, 2008).

Hence, both punishment and forgiveness help to erase a moral debt caused by a transgression and to restore a normal functioning, cooperative, relation between victim and transgressor in the wake of a moral transgression. In fact, punishment can sometimes promote subsequent forgiveness (Strelan and van Prooijen, 2013; Strelan et al., 2017). In such cases, punishment functions to have a moral debt either partially or fully repaid, and forgiveness then further absolves the perpetrator from further (potentially disproportional) suffering. However, in practice many transgressions get punished but not forgiven (Skitka and Bauman, 2008; Skitka, 2010) and some transgressions are forgiven but not punished (Leunissen et al., 2013; Van Houwelingen et al., 2018). This is likely because, arguably, very different calculations go into inflicting punishment or granting forgiveness. What determines whether organizational actors will punish or forgive a moral transgressor? In the following section we offer a self-regulatory account in terms of social cognitive theory.

## A Social Cognitive Analysis of Punishment and Forgiveness

Social cognitive theory was developed to explain the self-regulatory underpinnings of human behavior (Bandura, 1991, 2001, 2011). Without active self-regulation, people would not be able to set goals or strive for these goals and they could not respond to immediate contextual influences at best. According to social cognitive theory, a critical element of successful goal setting and goal striving is self-monitoring. This refers to paying adequate attention to one’s own behavior, the conditions under which it is enacted, and the (proximal and distal) effects it produces. This provides information for setting realistic goals and for evaluating progress towards these goals. Self-monitoring is not simply an automatic inspection of one’s actions; instead, preexisting cognitive structure and beliefs about the self (i.e., identity constructs) selectively influence which aspects of one’s functioning and surrounding context are attended to most.

Social cognitive theory views identity as a cognitive schema, stored in memory, that contains a set of beliefs and understandings one has of oneself (Bandura, 2002; Bandura and Bussey, 2004). Within that schema certain constructs can be more or less accessible, depending on one’s developmental history or other experiences (Bandura, 2011). Accessible constructs are considered to be ‘central’ to one’s self-concept, whereas less accessible constructs are considered to be ‘peripheral’ (Shao et al., 2008). According to social cognitive theory, people are motivated to behave in line with the beliefs and understandings they consider central (i.e., are more accessible; Bandura, 1991)—responses and behaviors that are not in line with those central beliefs are likely to result in aversive feelings, such as dissonance or guilt (Shao et al., 2008; Aquino et al., 2009).

Social cognitive theory is also relevant to the self-regulatory underpinnings of moral behavior. In dealing with morally loaded situations, people must extract, weight and integrate morally relevant information in the situation that confronts them. However, as all self-regulation, self-regulation of moral behavior builds on how cognitive schemas about the self (i.e., identity constructs) relate to the moral situation. The identity type in this respect that is directly relevant, from the perspective of social cognitive theory, is moral identity (Aquino et al., 2007, 2009, 2011; Reed et al., 2007; Boegershausen et al., 2015). As noted, moral identity refers to the centrality of moral beliefs to the self-concept (Bandura, 1991, 2002). The more moral beliefs are considered to be central to the notion of the self, the more acting in ways that are not in line with these beliefs will result in aversive and dissonantic feelings (Hoogervorst et al., 2010). In line with this reasoning, it has been shown that people high (vs. low) in moral identity centrality are more likely to describe themselves in moral terms (O’reilly and Aquino, 2011), they are motivated to engage in behaviors that are seen as morally commendable such as volunteering and other charitable behaviors, and they are less likely to engage in antisocial and unethical behaviors (Reynolds and Ceranic, 2007; Hertz and Krettenauer, 2016).

We argue that moral identity centrality also motivates responses to moral transgressions in a way that restores moral order. Arguably, the need for a reasserted moral order is stronger if one identifies more strongly with that order (i.e., when moral identity is central) then when this is less the case (i.e., when moral identity is peripheral). In fact, we argue that people for whom moral notions are central to the self-concept consider it to be more important to be part of a moral organizational community—i.e., a community of shared values, in which people are treated fairly and honestly, where leaders lead with integrity and so on—than people for whom morality is relatively peripheral to their notions of the self (Aquino et al., 2011; Tomlinson et al., 2014; Boegershausen et al., 2015). People with a central (vs. peripheral) moral identity, therefore, should also be more sensitive to intrusions against the moral order within the community (i.e., moral transgressions) and be more motivated to restore this order after such an intrusion.

As noted, research has identified two very different responses that can restore a sense of moral order: punishing or forgiving the transgressor (McCullough et al., 2010; Zheng et al., 2016, 2018; Strelan et al., 2017), depending upon the alignment between motive attributions for the response and motives for the transgression (Gollwitzer and Okimoto, 2021). Prior research has shown that a central (vs. peripheral) moral identity makes individuals more likely to forgive a transgressor (Aquino and Reed, 2002; Aquino et al., 2007). However, other research has shown that a central moral identity is associated with higher levels of punitiveness (Hofmann et al., 2018). In sum, existing research does not tell us if or when a central (vs. peripheral) moral identity motivates punishment of moral transgressions, and when it will lead to forgiveness.

Based on social cognitive theory, we argue that a crucial factor that drives whether people with a central (vs. peripheral) moral identity punish or forgive a moral transgression is rooted in the nature of the transgression itself, specifically with whether or not a moral norm was transgressed intentionally. Social cognitive theory argues that “in dealing with moral dilemmas, people must, therefore, extract, weight, and integrate the morally relevant information in the situations confronting them” (Bandura, 1991, p. 69). Intentionality is crucial morally relevant information in the decision to punish or forgive a transgression. This is because intentional transgressions signal that the transgressor was aware of the relevant moral norms, but chose to ignore them (Kim et al., 2006). Unintentional transgressions signal the reverse: a transgressor was unaware of the relevant norms to uphold. Punishment in response to a moral transgression serves to have a perpetrator repay a moral debt, and, by that, communicates that the moral order is still intact. Unintentional transgressors, in contrast, may simply need being made aware of the existence of a norm to mend their ways. In such cases, punishment may actually be perceived as disproportional, or even as a moral transgression itself. This clearly undercuts its potential to effectively restore a sense of moral order. However, even unintentional transgressions typically cause harm. Hence, while unintentional transgression typically do not meet the standards for punishment deservingness, they need to be forgiven for both the victim and the transgressor to be able to move on (Fitness, 2001). These arguments culminate in our hypotheses:

*Hypothesis 1*: Intentional (vs., unintentional) transgressions are more likely to be punished, especially when the responding person is high (vs., low) in moral identity centrality.

*Hypothesis 2*: Unintentional (vs., intentional) transgressions are more likely to be forgiven, especially when the responding person is high (vs., low) in moral identity centrality.

## Study Overview

We conducted a laboratory experiment (Study 1) and a field study among organizational members with a supervisory role (Study 2). We manipulated moral identity centrality in Study 1 and measured it in Study 2. In Study 1, we included a behavioral (rather than a self-report) transgression response. This meant that we could measure punishment, but not forgiveness. Hence, in Study 1 we tested Hypothesis 1 only. In Study 2, we asked leaders in organizational settings to recall an episode in which a follower had committed a transgression that was either intentional or unintentional. We then measured their punitive and forgiving responses using self-report measures. This allowed testing Hypothesis 1 and 2.

### Study 1

#### Method

##### Design and Participants

We assigned participants randomly to one of the four conditions that resulted from orthogonally manipulating transgression intentionality (intentional vs. unintentional) and moral identity centrality (high vs. low). As we will explain in more detail, our dependent variable was a count—we thus set out to estimate a binomial regression model (i.e., Poisson regression, or negative binomial regression, depending on the level of dispersion). We used the *sizePoisson* function from the R-package PowerMediation (Qiu and Qiu, 2021) to estimate that we needed 83 participants to estimate a medium-sized interaction effect (i.e., *f* = 0.25) with *B* = 0.8 and *α* = 0.05. We ran this study for 1 week in the lab of a Dutch business school among undergraduate students. From experience we knew to expect between 100 and 150 participants during a week. This way, we recruited 112 undergraduate students who participated in exchange for course credits. Sensitivity analysis (Faul et al., 2007), using the *powerPoisson* function from the same R-package (Qiu and Qiu, 2021), indicates that our design with 112 participants with *α* = 0.05 and *B* = 0.91 was sufficiently powered to detect a medium-sized interaction effect (i.e., *f* = 0.25). Of the included participants, 60 were male and 52 were female (*M*_age_ = 20.32, *SD* = 2.05).

##### Procedure

On arrival, we informed participants that they would participate in two unrelated studies: a handwriting task and a negotiation study. The first study was the moral identity centrality manipulation taken from Aquino et al. (2009). In the high moral identity salience condition, we asked participants to first copy the nine traits that comprise Aquino and Reed’s (2002) moral identity instrument (Caring, Compassionate, Fair, Friendly, Generous, Helpful, Hardworking, Honest, and Kind) on a sheet of paper. In the low moral identity salience condition, we asked participants to copy nine positively valanced traits that are unrelated to moral content (Carefree, Compatible, Favorable, Generally, Happy, Harmless, Open-Minded, Respectable, and Polite). Afterwards, we asked all participants in both conditions to write a short paragraph about themselves using the trait words they had copied. Copying the moral identity traits and using them in a paragraph has been shown to be effective in making moral identity salient (Aquino et al., 2007, 2009; Thau et al., 2007).

Upon finishing the handwriting task, participants learned that they would be paired with another person in the lab, with whom they would engage in a computer-mediated interaction. Furthermore, we told them that one of them would be appointed as the leader and the other as the follower based on their responses to a questionnaire they were required to fill out at the start of the experiment. This questionnaire was said to measure leadership skills. We did this to set up a relationship between our participants and to make decisions on punitive responses appear to be a natural part of the participant’s role. Next, participants played a modified version of the trust game (Berg et al., 1995). In the trust game we used in this study, the leaders learned they would be allocated an amount of € 10 (about $ 13 dollar at the time) which they could decide to invest in their follower in the form of tickets (1 ticket = € 1). Any amount they invested would be tripled. Hence, if the leader invested € 10 in the follower, the follower would receive € 30 (about $ 40). To ensure that most leaders would invest in their subordinate we informed them that if they kept the money, they would receive half of this amount (€ 5 or $ 6.50). It was then up to the follower to decide how much (from € 0 to € 30) s/he would return to the leader. Finally, participants learned that they would play multiple rounds of the game and that they would be the allocator in every round.

We then asked three questions (i.e., “How many tickets do you initially receive?,” “If you invest in your subordinate, how many tickets does the latter receive?,” “You have invested 10 euros and your subordinate has decided to return half of the amount s/he received. How much did you get?”). We asked these questions at this point to assess whether the rules of the interaction task were clear to participants, and we corrected them when one or more of their answers were incorrect. These questions also allowed us to manipulate transgression intentionality (see below).

Next, participants had to choose whether they wanted to invest € 10 in their follower. All decided to do so. Within a minute, leaders learned that they received € 5 in return—this constituted the follower’s transgression. To check whether the participants had read this information, we asked them to type how many Euros they had received. All participants correctly indicated they had received 5 euros.

After these questions, we introduced the violation type manipulation. The follower e-mailed a private message to the leader. In unintentional transgression condition, participants read:

*Hey, I did not really understand the rules, but I gave you 10/2 = 5 euros.*

In contrast, in the intentional violation condition, participants read:

*Hey, I received 10 euros, so I gave you 10/2 = 5 euros.*

To support this manipulation, in the intentional transgression condition participants learned that their follower had answered all questions correctly and thus had a clear understanding of the interaction task at hand. In the accidental transgression condition, participants learned that their follower had only one correct answer.

Afterwards, we solicited the dependent measure. We then informed participants that no further rounds would be played. Finally, we thanked and debriefed participants.

##### Manipulation Check

To assess whether our transgression intentionality manipulation was successful, we asked participants whether they believed the follower acted intentionally when making their decision on how much money to share (1 = *strongly disagree*; 7 = *strongly agree*).

##### Dependent Variable

We informed participants that, because of their leader role they could claim money back from their follower (from zero to a maximum of 25 Euros). We used the number of euros taken as an index of punishment.

#### Results

##### Manipulation Check

ANOVA on the check of the intentionality manipulation revealed a significant main effect of transgression intentionality, *F*(1, 99) = 15.36, *p* < 0.001, $\eta_{p}^{2}$ = 0.13. Participants in the high intentionality condition believed that their interaction partner acted more intentional than participants in the low intentionality condition (*Ms* = 5.28 vs. 4.27, *SDs* = 1.27 and 1.47 respectively). Neither the main effect of moral identity *F*(1, 99) = 1.53, *p* = 0.220, $\eta_{p}^{2}$ = 0.02, nor the interaction effect was significant *F*(1, 99) = 0.50, *p* = 0.481, $\eta_{p}^{2}$ = 0.01.

##### Punishment

**Figure 1:**
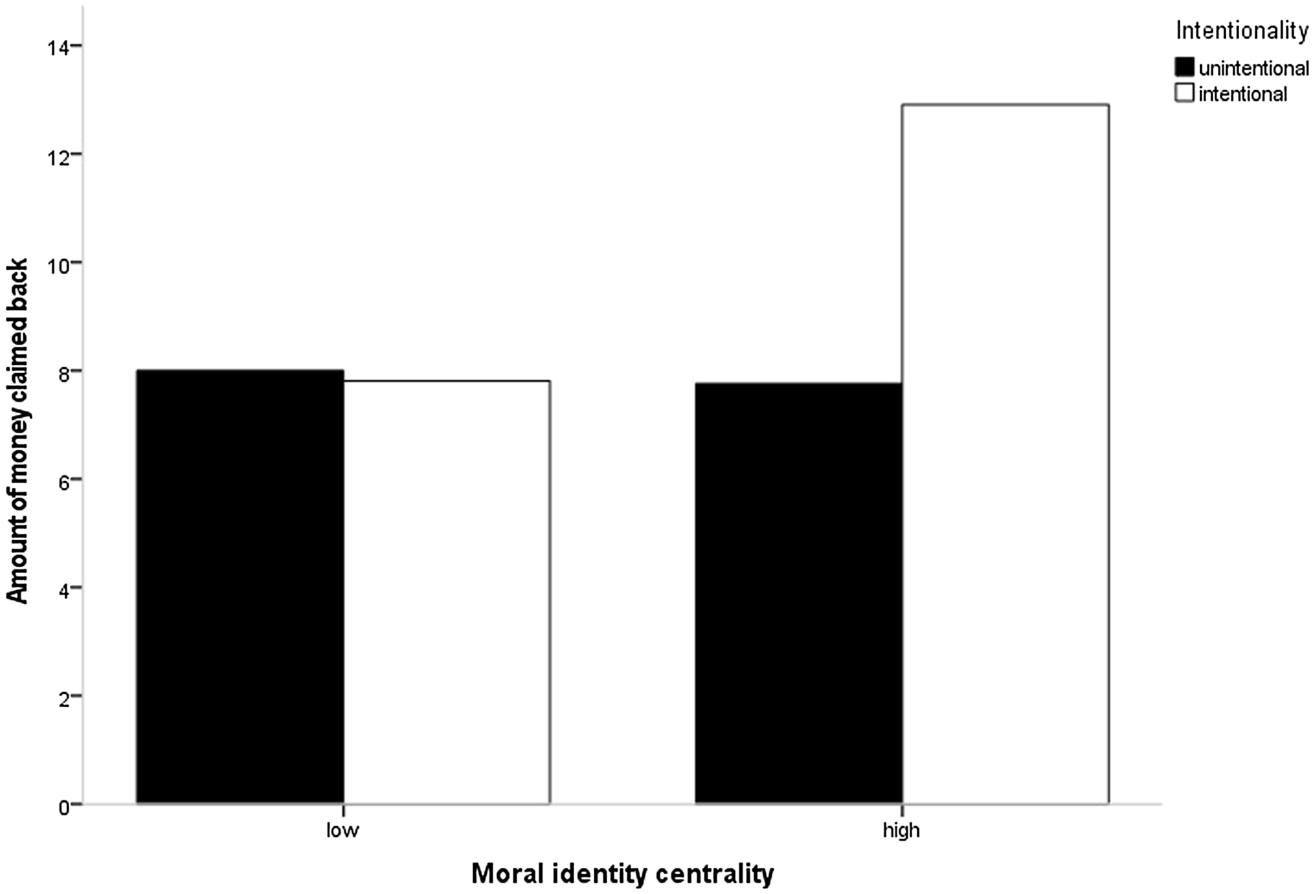
Interaction between moral identity salience and transgression intentionality on the amount of money claimed back in Study 1.

An overdispersion test revealed significant overdispersion in our data, *ratio* = 3.91, *χ*^2^ = 421.99, *p* < 0.001, as such we estimated a negative binomial regression model (Ver Hoef and Boveng, 2007; Hilbe, 2011) with euros taken away as criterion and with transgression intentionality and moral identity and the interaction between the two as predictor variables. This model revealed significant main effects of transgression intentionality, *b* = −0.59, *SE* = 0.21, *z* = −2.84, *p* = 0.005, *IRR* = 0.55, and moral identity *b* = −0.63, *SE* = 0.21, *z* = −2.97, *p* = 0.003, *IRR* = 0.53. As predicted, the analysis revealed a significant Transgression Intentionality × Moral Identity effect, *b* = 0.51, *SE* = 0.13, *z* = 3.99, *p* < 0.001, *IRR* = 1.67 (see Figure 1).^1^

Simple effects tests revealed that when moral identity was salient, participants punished an intentional transgression significantly harsher than an unintentional transgression (*M* = 12.91 vs. *M* = 7.76, *SD* = 6.18 vs. *SD* = 5.76, respectively, *b* = −0.43, *SE* = 0.09, *z* = −5.00, *p* < 0.001, *IRR* = 0.65). However, when moral identity was not salient, there was no significant difference in punitive behavior (*M* = 7.81 vs. *M* = 8.00, *SD* = 5.67 vs. *SD* = 5.23, respectively, *b* = 0.08, *SE* = 0.09, *z* = 0.84, *p* = 0.40, *IRR* = 1.08).

From a different vantage point, a salient (vs. non-salient) moral identity led to harsher punishment of intentional transgressions (*M* = 12.91 vs. *M* = 7.81, *SD* = 6.18 vs. *SD* = 5.67, respectively, *b* = −0.39, *SE* = 0.08, *z* = −4.73, *p* < 0.001, *IRR* = 1.13) but not of nonintentional transgressions (*M* = 7.76 vs. *M* = 8.00, *SD* = 5.76 vs. *SD* = 5.23, respectively, *b* = 0.12, *SE* = 0.10, *z* = 1.22, *p* = 0.224, *IRR* = 0.67).

### Discussion of Study 1 and Introduction to Study 2

The findings of Study 1 support Hypothesis 1, in showing that moral identity centrality causes people to distinguish more clearly between intentional and unintentional transgressive behavior in their punitive response. We obtained this support using an established experimental paradigm to test behavioral reactions to transgressive behavior (the trust game).

Our reliance on the trust game provided Study 1 with a high degree of internal validity. However, a drawback of our procedure is that we could not formally test Hypothesis 2 (about forgiveness). Furthermore, because Study 1 was a laboratory experiment, another limitation is that we could not test our prediction among those with a supervisory role in actual organizations. In particular, our respondents did not appear to be particularly punitive, given that the highest condition-mean was roughly at the midpoint of possible punishment. To address these limitations, we designed Study 2. We conducted Study 2 on Amazon Mechanical Turk (AMT), which allowed us to include participants with a supervisory role in the organization that they worked for. AMT has been shown to be an effective way to recruit organizational members with a supervisory or leadership role (Van Houwelingen et al., 2017). Finally, we included both punishment and forgiveness as outcome variables.

### Study 2

#### Method

##### Design and Participants

Moral identity centrality was a continuous, between-subjects variable. Transgression intentionality was a 2-level between-subjects factor (high. vs. low). We invited 250 participants. Of those invited, 221 could be included in the data analyses (see: Procedure and participant inclusion for details). Sensitivity analysis (Faul et al., 2007) indicates that our design with 221 participants with *α* = 0.05 and power = 0.95 was sufficiently powered to detect a medium-sized interaction effect (i.e., *f* = 0.24). To be allowed to participate, participants had to hold paid employment at the time of the study and had to supervise at least one other employee. Of the participants, 65% were male and 35% were female. The mean age was 34.43 (*SD* = 10.30). On average, participants had worked for 7.08 years (*SD* = 5.22) in their organization, and for 4.20 years (*SD* = 3.36) in their current position. Eight percent of participants had primary education (high school) as highest completed education, 22% had some college, 10% had an associate degree, 44% had a bachelor degree, 13% a master degree, and 3% a doctoral or professional degree (PhD, JD, or MD).

##### Procedure and Participant Inclusion

We based our proceedings on Leunissen et al. (2013) as well as Van Houwelingen et al. (2018). Specifically, participants in the unintentional transgression condition read:

“*In this study, we are interested in social experiences at the workplace. Please recall (remember) a situation at work in which one of your subordinates unintentionally or accidentally committed a transgression. We would like to ask you to describe a situation in which one of your subordinates committed such a transgression and did so unintentionally or accidentally. Please describe this situation in 3–5 sentences.*”

Participants in the intentional transgression condition read:

“*In this study, we are interested in social experiences at the workplace. Please recall (remember) a situation in which one of your subordinates intentionally committed a transgression. We would like to ask you to describe a situation in which one of your subordinates committed such a transgression and did so intentionally. Please describe this situation in 3–5 sentences.*”

Subsequently, we introduced our dependent measures. Upon finishing, we thanked participants for their participation.

A research assistant, unaware of the study aims or hypotheses, evaluated if each of the recollections adhered to the instructions. Based on this, we removed 29 cases that did not describe a transgression committed by a subordinate (but by a colleague of the same rank, or by a peer, outside of the work setting), that described an intentional transgression in the condition where we asked to describe an unintentional transgression (or vice versa), or where the recollection did not describe a transgression.

##### Measures

Participants responded to all measures on a seven-point Likert-scale (1 = *strongly disagree*, 7 = *strongly agree*).

We measured moral identity centrality using the 10-item moral identity measure developed by Aquino and Reed (2002). We presented participants with the same nine moral adjectives we used in the moral identity centrality prime in Study 1. Subsequently, participants rated to what extent these adjectives are an important part of their own identity. This measure consists of the 5-item *internalization* scale (e.g., “Being someone who has these characteristics is an important part of who I am”) and the 5-item *symbolization* subscale (e.g., “I am actively involved in activities that communicate to others that I have these characteristics”). Internalization represents the extent to which moral traits are imbedded in one’s self-concept; symbolization represents the extent to which one expresses moral behaviors through one’s public actions. Our hypotheses pertain to the self-relevance of morality, or in other words, to the internalization subscale. Yet, we measured symbolization in addition to internalization, for exploratory purposes: A significant influence of moral identity symbolization may be taken to indicate that participants may also engage in punitive and forgiving behavior to signal their moral identity to others.

We checked the *intentionality manipulation* with one item: “In the situation you just described, I feel that my employee intended for this to happen.”

We measured *punishment* with a 3-item disciplinary action scale, adapted from Rosen and Jerdee (1974). The three items describe the disciplinary actions most commonly used in organizations (Beyer and Trice, 1984). We asked participants to what extent they believed the following actions are the appropriate response to the situation they described: “To give a written reprimand to this employee,” “To suspend this employee,” and “To discharge this employee.”

We measured *forgiveness* with a 3-item scale adapted from Aquino et al. (2007). Specifically, we asked to what extent respondents agreed with “I had trouble forgiving this employee,” “I found it difficult to put aside negative feelings about this employee,” and “I found it difficult to let go of my resentment to this employee.” We recoded these items in a forgiveness scale.

#### Results

Table 1 presents the means, standard deviations, Cronbach’s alpha coefficients and correlations between the Study 2 variables.

**Table 1:** Descriptive statistics, correlations, and reliabilities, Study 2.

Variables	*M*	*SD*	1	2	3	4	5
1. Transgression intentionality	−0.01	1.00					
2. Moral identity internalization	6.13	0.92	0.01 (0.912)	0.86			
3. Moral identity symbolization	4.42	1.34	−0.00 (0.969)	0.29 (<0.001)	0.90		
4. Forgiveness	5.03	1.81	−0.41 (<0.001)	0.14 (0.037)	−0.01 (0.917)	0.97	
5. Punishment	3.32	1.69	0.37 (<0.001)	0.04 (0.606)	0.01 (0.855)	0.58 (<0.001)	0.77

*N = 221. Table presents means and standard deviations, correlations and p values of the correlations (within brackets). Cronbach’s alpha coefficients are presented at the main diagonal. Intentionality was effect coded with −1 presenting unintentional transgressions and 1 intentional transgressions*.

##### Violation Type Check

An independent samples t-test revealed that our manipulation of intentionality was successful *t*(219) = −17.16, *p* < 0.001. Respondents in the intentional violation condition believed the transgressing employee to have acted more intentionally than respondents in the unintentional condition (*Ms* = 5.87 vs. 2.35, *SDs* = 1.55 and 1.51 respectively).

##### Punishment

**Figure 2:**
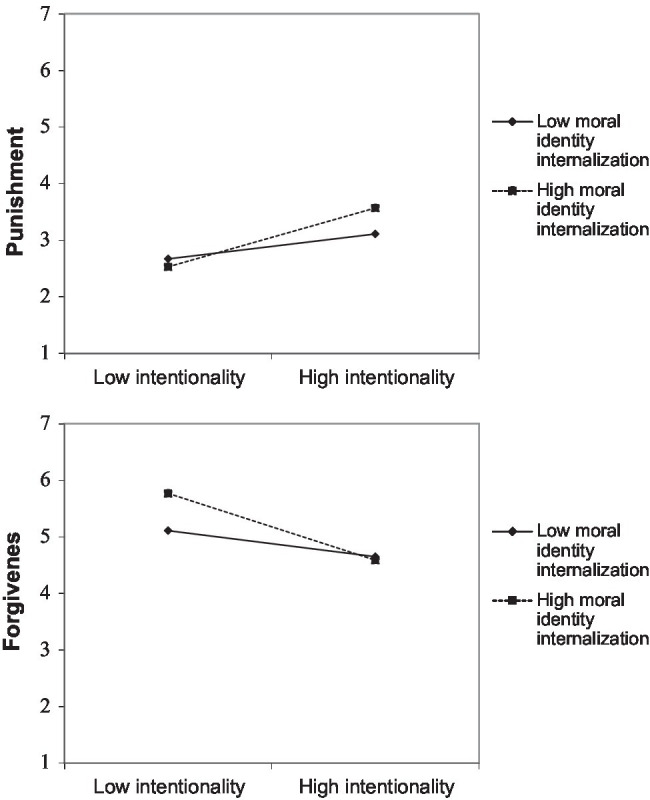
Interaction between moral identity salience and transgression intentionality on punishment (top panel) and forgiveness (lower panel) in Study 2.

Table 2 presents the results. As hypothesized, we found a significant Transgression Intentionality × Moral Identity Internalization interaction effect (see Figure 2). Simple slopes analyses (Aiken et al., 1991) revealed that leader’s moral identity internalization predicted punishment of intentional transgressions (*β* = 0.18, *t* = 1.93, *p* = 0.055) but not of unintentional transgressions (*β* = −0.12, *t* = −1.32, *p* = 0.189). From a different vantage point, for leaders with a high moral identity internalization (1 *SD* above the mean) the effect of intentionality on punishment was (*β* = 0.51, *t* = 5.84, *p* < 0.001) than for leaders with a low moral identity internalization (*β* = 0.22, *p* = 0.016). We did not find significant intentionality by moral identity symbolization interaction effects.

**Table 2:** Regression results of Study 2.

**Dependent variable**	**Punishment**	**Forgiveness**
**Step 1**, *R^2^*, *R^2^_adj_*	0.14 (<0.001) 0.13	0.19 (<0.001) 0.18
Intentionality	0.37, 5.91 (<0.001)	−0.41, −6.65 (<0.001)
Moral identity internalization	0.03, 0.47 (0.640)	0.16, 2.49 (0.017)
Moral identity symbolization	−0.00, 0.07 (0.948)	−0.05, −0.85 (0.396)
**Step 2**, *R^2^, R^2^_adj_, ΔR^2^*	0.16 (<0.001), 0.14, 0.02 (0.07)	0.22 (<0.001), 0.20, 0.03 (0.012)
Intentionality (TI)	0.37, 5.94 (<0.001)	−0.41, −6.75 (<0.001)
Moral identity internalization (MII)	0.03, 0.43 (0.668)	0.15, 2.41 (0.017)
Moral identity symbolization (MIE)	−0.01, −0.19 (0.849)	−0.04, −0.61 (0.543)
TI × MII	0.15, 2.30 (0.023)	−0.18, −2.83 (0.005)
TI × MIE	−0.02, −0.35 (0.724)	0.11, 1.76 (0.079)

*N = 221; Table presents β coefficients, t values, and two-sided p values (in round brackets)*.

##### Forgiveness

Table 2 presents the results. As hypothesized, we found a significant Transgression Intentionality × Moral Identity Internalization interaction effect (see Figure 2). Simple slopes analyses (Aiken et al., 1991) revealed that leader’s moral identity internalization predicted relationship restauration after an unintentional transgression (*β* = 0.33, *t* = 3.69, *p* < 0.001) but not after an intentional transgression (*β* = −0.03, *t* = −0.30, *p* = 0.768). From a different vantage point, leaders with a high moral identity (1 *SD* above the mean) were less likely to forgive after an intentional than an unintentional transgression (*β* = −0.58, *t* = −6.78, *p* < 0.001). For leaders with a low moral identity internalization (1 *SD* above the mean) this effect of intentionality was weaker (*β* = −0.23, *t* = −2.61, *p* = 0.010). Again, we did not find significant moral identity symbolization by transgression intentionality interaction effects.

##### Supplemental Analyses

Recalled transgressions that were intentional may have been more severe than unintentional transgressions, and this difference in severity may be responsible for the differential tendencies to forgive or punish intentional (vs. unintentional) transgressions, as a function of moral identity. To evaluate this possibility, a coder unaware of the study’s hypotheses coded the recalled transgressions in terms of severity (1 = *not severe at all*, 5 = *very severe*). Initial analyses showed that intentional transgressions were indeed more severe than unintentional transgressions (*β* = 0.24, *t* = 3.65, *p* < 0.001). We therefore conducted the same analyses as reported above, but this time we included (in addition to the main effects of transgression intentionality, moral identity internalization and moral identity symbolization, and the Transgression Intentionality × Moral Identity Internalization and Transgression Intentionality × Moral Identity symbolization interactions) also the main effect of transgression severity and the Transgression Severity × Moral Identity Internalization and the Transgression Severity × Moral Identity Symbolization interactions. These analyses showed that more severe transgressions were more likely to be punished (*β* = 0.35, *t* = 5.78, *p* < 0.001) and less likely to be forgiven (*β* = −0.23, *t* = −3.75, *p* < 0.001). However, transgression severity did not interact with moral identity internalization to influence punishment (*β* = −0.11, *t* = −1.65, *p* = 0.101) or forgiveness (*β* = 0.08, *t* = 1.21, *p* < 0.227). Transgression severity also did not interact with moral identity symbolization to influence punishment (*β* = −/06, *t* = −0.97, *p* = 0.333) or forgiveness (*β* = 0.04, *t* = 0.67, *p* = 0.507). Moreover, in these analyses, the Transgression Intentionality × Moral Identity Internalization interaction still significantly predicted punishment (*β* = 0.14, *t* = 2.16, *p* = 0.032) and forgiveness (*β* = −0.17, *t* = −2.65, *p* = 0.009). In sum, the effects of transgression intentionality cannot be reduced to differences in transgression severity.

## General Discussion

Across two studies, we found that moral identity uniquely shapes punitive and forgiving responses to transgressors of moral norms. Specifically, people high (vs., low) in moral identity centrality were more (vs., less) likely to punish intentional (vs., unintentional) transgressions severely. However, unintentional, rather than intentional transgressions, were more likely to be met by forgiving responses among people high (vs., low) in moral identity centrality. We obtained these effects in samples taken from different populations (business students in Study 1; organizational supervisors in Study 2), using different research methodologies (a laboratory experiment and a field study) and by operationalizing our key variables in various ways (i.e., situationally induced vs. dispositional moral identity centrality, manipulated vs. recalled follower misconduct, and currently measured vs. recalled punishment behavior and forgiveness). This methodological diversity bolsters confidence in our conclusions (Campbell and Fiske, 1959).

### Theoretical Implications

Organizational scholars have studied punishment for decades (Luthans and Kreitner, 1973; Arvey and Ivancevich, 1980; Ball et al., 1992; Podsakoff et al., 2006; McNamara et al., 2022). However, almost all of this research took an instrumental approach by studying the performance-enhancing (and performance-undermining) effects of punishment (Ross, 1975; Podsakoff et al., 2006). A much smaller number of studies has zoomed in on punishment as a response to moral transgressions in organizations. Yet even this research assumed an instrumental focus, showing that if the leader or the organization gained from a transgression, then he/she was inclined towards less severe punishment (Hoogervorst et al., 2010; Cramwinckel et al., 2013; Desmet et al., 2015; Van Houwelingen et al., 2017). However, moral transgressions do threaten the moral order within an organization (Strelan and van Prooijen, 2013). It is thus important to develop a moral perspective on punishment of moral transgressions. By studying the effects of moral identity and transgression intentionality on punishment (and forgiveness) within the context of social cognitive theory, our paper presents a step towards understanding the morality of punishment in organizations.

Perhaps because much research has taken an instrumental view of the uses of punishment in organizational contexts, most studies seem to assume that the antecedents (e.g., below-par performance) and intended consequences (e.g., inciting better performance) of punishment are, if not the same, then still very closely related (Podsakoff et al., 2006). This may be true for the performance-context but is arguably not the case for the moral aspect of punishment. Our studies illustrate that moral identity centrality drives punishment in a way that is theoretically as well as practically distinguishable from the consequences of punishment. Hence, to understand the moral connotations and aspects of punishment within organizational consequences, it is imperative to not only focus on the consequences, but also on the antecedents of punishment behavior (Hoogervorst et al., 2010).

Whereas punishment has received much attention from organizational scholars, forgiveness has only recently come into focus of organizational research (Fehr and Gelfand, 2012; Zheng et al., 2016, 2018). Work on forgiveness in organizational contexts often takes a prescriptive approach (Ferch and Mitchell, 2001; Caldwell and Dixon, 2010), typically conceptualizing forgiveness as a personal or organizational virtue (Kurzynski, 1998). As such, our focus on the antecedents that forgiving behavior within organizations shares with punishing behavior constitutes one of the first attempts to flesh out the motivational underpinnings of organizational forgiveness. Our research indicates that forgiveness, like punishment, can be meaningfully conceptualized as a way in which organizational actors can maintain moral order within their community—this is in line with a recent research stream in social psychology that emphasizes the functional similarity between both responses (see Strelan, 2017, for an overview of this literature).

Lastly, our research is relevant to social cognitive theory of moral identity (Bandura, 2014). More specifically, the relation between moral identity centrality and responses to moral transgressions has not been deeply explored (Shao et al., 2008). Some studies have found positive relation between moral identity centrality and forgiveness, and a negative relation with punishment (Aquino and Reed, 2002; Aquino et al., 2007). These studies were conducted, however, in relation to quite unusual events (i.e., the 9/11 terrorist attacks) and similarly unusual targets of forgiveness and punishment (i.e., perpetrators of those attacks) with whom the participants did presumably not have cooperative relationships. In addition, particularly the punitive measures that these studies focused on were quite extreme (e.g., bombing the hiding places of the planners of the attacks). The model we present here associates moral identity centrality both with a stronger propensity to punish as well as to forgive, depending on the type of the offense. As such, we show that a central (*vs* peripheral) moral identity can make people more punitive and more forgiving, depending on the type of transgression they are confronted with. The reason for this is, we argue, that moral norms and principles are used to determine punishment and forgiveness deservingness, so that people with a central (*vs* peripheral) moral identity are prepared to respond punitively to moral transgression if that is required by the moral principles they apply to the situation.

### Practical Implications

A first implication of our research is that it underscores how the role of organizational members high in moral identity centrality is vital for organizational sociality. Punishment and forgiveness as responses to moral transgressions play a crucial role in social maintenance within an organizational community (Gollwitzer and Okimoto, 2021). If left unchecked (i.e., neither punished nor forgiven) moral transgressions may quickly erode justice perceptions as well as perceptions of the moral climate within the organization (Ball et al., 1992; Skarlicki and Kulik, 2004). Hence, the presence of people within an organizational community to address the moral failings of others in some way (i.e., either punitively or forgivingly) is of crucial importance for the moral climate, ethical culture and sense of justice within that community (Rai and Fiske, 2011; Joshi and McKendall, 2016; Heiphetz et al., 2018). Our studies show these people are likely those for whom their moral identity is central to their self-concept. Our studies show how moral identity centrality is the motivational basis underpinning both punishment and forgiveness behavior within organizations.

Moral identity centrality is a state and a trait (Shao et al., 2008; Aquino et al., 2009). That is to say: Even though moral identity centrality is rooted in personality, it can—as illustrated in Study 1—also be situationally induced (Weaver, 2006). Hence, organizations can influence the centrality of morality to the self-concept of employees in two main ways. First, organizations can make moral identity centrality a factor in their recruiting process so that new recruits are likely to be high in moral identity centrality. Secondly, and perhaps more importantly, they can emphasize moral norms and values in their communication with employees as well as in decision making procedures. This is likely to prime moral identity centrality in a roughly comparable way as our prime in our Study 1 primed moral identity centrality for our participants. In the long run, consistent corporate policy in this respect is likely to affect even those employees for whom morality was less than central to their identity when joining the organization. Social cognitive theory suggests that concepts can become more central to the self-concept (i.e., accessible) the more people rely on them in their daily life (Bandura, 1991). Hence, an organization that consistently emphasizes morality in their operations is likely to induce more moral identity centrality, even among employees who originally had more peripheral moral identities.

### Limitations and Suggestions for Future Research

As all research, ours comes with limitations that should be discussed. One limitation is that we focused on short-term one-off interactions between strangers in Study 1. Relations in organizations tend, of course, to be more long-term and between people that do know each other. We therefore conducted Study 2, a field study, to investigate the effects we found in Study 1 in an ecologically valid sample. We found converging evidence for our predicted effects in Study 2 as well. In Study 2 we controlled for transgression severity as an alternative explanation for the role of transgression intentionality. However, it remains possible that there were other differences between recalled intentional and unintentional transgressions that we did not account for in this study. Therefore, the controlled setting of Study 1, which allowed avoiding confounds of transgression intentionality makes the evidence that emerged from this study important for the robustness of our conclusions. This suggests that the effects we identified in the lab translate to the field.

In all, more research is needed that can connect the realism that is needed to study the many aspects of punishment and forgiveness with the rigor that these phenomena deserve. Combining laboratory studies with mundane realism with field samples is one way to approach this, but one could for instance also consider mixed-methods designs in which qualitative and quantitative approaches are combined. For example, an interesting approach would be to study, first, the interpersonal scripts involved in naturally occurring instances of either punishment or forgiveness in organizations, and then to use survey-based research or even experimental methods to confirm propositions derived from this first part (Fitness and Peterson, 2008). Studies of interpersonal revenge and forgiving in close relationship may in that case well serve as an inspiration and a yardstick (McCullough et al., 1997; Finkel et al., 2002).

One interesting additional research question that emerges from our work, for instance, is how transgression intentionality and moral identity centrality relate to other (i.e., non-punitive and non-forgiving) possible responses to transgressions (e.g., acting indifferently; McClelland, 2020). In our studies, we intentionally restricted the options our participants had to punitive (in Studies 1 and 2) and forgiving (Study 2) responses, but in reality, people obviously have more options. Extending the behavioral choice set would therefore be a good way to increase the mundane realism of studies into the phenomena under study here.

While in the organizational literatures, punishment and forgiveness have predominantly been studied separately, scholars studying the Valuable Relationship Hypothesis (which comes from evolutionary scholarship) have often studied both types of responses in conjunction (e.g., McCullough, 2008; Burnette et al., 2012; Ohtsubo and Yagi, 2015). The Valuable Relationship Hypothesis implies that people have an instinct for both punishment and forgiveness in response to transgressions of others which is rooted in an assessment of maintaining a relationship with a transgressor: People punish when the value of deterrence is higher than the value of continuing the relationship, and forgive otherwise (McCullough et al., 2010). Over time, these instincts may have become formalized in moral preferences (Tabak et al., 2012). Our results are broadly in line with this hypothesis: According to the Valuable Relationship Hypothesis transgression intentionality determines the value of deterrence and should therefore inspire punishment (vs. forgiveness; McCullough, 2008). However, most of the empirical evidence for this hypothesis comes from non-formal groups or even from the study of higher-order animal groups (e.g., apes; McCullough et al., 2010). Hence, our paper provides first intriguing evidence that the Valuable Relationship Hypothesis may also apply in more formal work settings. Future research may further explore this possibility.

Last, recent scholarship has started to suggest that punitive and forgiving responses are most likely to be effective in restoring order in cases where the attributed motives for these responses are in line with motives that underlay the original transgression (Strelan et al., 2020; Gollwitzer and Okimoto, 2021). For instance, punishment is supposed to be more (vs., less effective) in cases where a transgression set out to cause harm and the punishment is attributed to the motive to restore that harm (Strelan, 2017) Our research shows that people whose moral identity is central (vs., peripheral) to their sense of self, are more likely to clearly distinguish between intentional and unintentional transgressions when deciding on punitive and forgiving responses. An emerging hypothesis, therefore, is that moral identity centrality (vs., peripherality) may be related to the enactment of punishment and forgiveness that is better aligned with transgressor motives. Testing this hypothesis is an exciting opportunity for further research.

## Concluding Remarks

Moral transgressions have the potential to affect the moral fabric of organizational communities. Punishment and forgiveness of such moral transgressions are the two tools that community members have to maintain and restore that moral fabric. The function, role, antecedents and consequences of moral punishment and forgiveness therefore deserve more research attention. We hope that our research stimulates future work that studies punishment and forgiveness in organizations simultaneously.

## Data Availability Statement

The raw data supporting the conclusions of this article will be made available by the authors, without undue reservation.

## Ethics Statement

The studies involving human participants were reviewed and approved by RSM Internal Review Board Section Experimental Research, Rotterdam School of Management. The patients/participants provided their written informed consent to participate in this study.

## Author Contributions

GH and MD contributed to the manuscript equally. NH provided research idea. LM and DC provided feedback and guidance. All authors contributed to the article and approved the submitted version.

## Conflict of Interest

The authors declare that the research was conducted in the absence of any commercial or financial relationships that could be construed as a potential conflict of interest.

## Publisher’s Note

All claims expressed in this article are solely those of the authors and do not necessarily represent those of their affiliated organizations, or those of the publisher, the editors and the reviewers. Any product that may be evaluated in this article, or claim that may be made by its manufacturer, is not guaranteed or endorsed by the publisher.

## Footnotes

^1^This interaction effect was still significant when we treated our outcome variable as continuous and used ANOVA: *F*(1, 108) = 4.58, *p* = 0.035, $\eta_{p}^{2}$ ￼ = 0.04.
